# Wetware network-based AI: a chemical approach to embodied cognition for robotics and artificial intelligence

**DOI:** 10.3389/frobt.2025.1694338

**Published:** 2026-01-05

**Authors:** Luisa Damiano, Antonio Fleres, Andrea Roli, Pasquale Stano

**Affiliations:** 1 Research Center for Complex Systems (CRiSiCo), Department of Communication, Arts and Media, IULM University, Milan, Italy; 2 Department of Computer Science and Engineering (Campus of Cesena), Università di Bologna, Cesena, Italy; 3 European Centre for Living Technology, Venice, Italy; 4 Department of Biological and Environmental Sciences and Technologies (DiSTeBA), University of Salento, Lecce, Italy

**Keywords:** wetware network-based artificial intelligence (WNAI), wetware neuromorphic engineering, embodied AI, chemical networks, chemical robotics, minimal artificial agents, autonomy, network cybernetics

## Abstract

*Wetware Network-Based Artificial Intelligence* (WNAI) introduces a new approach to robotic cognition and artificial intelligence: autonomous cognitive agents built from synthetic chemical networks. Rooted in *Wetware Neuromorphic Engineering*, WNAI shifts the focus of this emerging field from disembodied computation and biological mimicry to reticular chemical self-organization as a substrate for cognition. At the *theoretical level*, WNAI integrates insights from network cybernetics, autopoietic theory and enaction to frame cognition as a materially grounded, emergent phenomenon. At the *heuristic level*, WNAI defines its role as complementary to existing leading approaches. On the one hand, it complements embodied AI and xenobotics by expanding the design space of artificial embodied cognition into fully synthetic domains. On the other hand, it engages in mutual exchange with neural network architectures, advancing cross-substrate principles of network-based cognition. At the *technological level*, WNAI offers a roadmap for implementing chemical neural networks and protocellular agents, with potential applications in robotic systems requiring minimal, adaptive, and substrate-sensitive intelligence. By situating wetware neuromorphic engineering within the broader landscape of robotics and AI, this article outlines a programmatic framework that highlights its potential to expand artificial cognition beyond silicon and biohybrid systems.

## 1 Research rationale and programmatic orientation

This article introduces Wetware Network-Based Artificial Intelligence (WNAI) as a theoretical framework (perspecting future experimental implementations) for developing substrate-sensitive architectures for artificial cognition, grounded in chemical systems and relevant to the advancement of robotics and AI. WNAI has a twofold origin. On one side, it is rooted in the legacy of network cybernetics, that is, a foundational tradition in networked AI initiated by Warren McCulloch and Walter Pitts, with theoretical filiations in autopoiesis and enaction (Section 3). On the other side, it is grounded in recent advances in synthetic biology (SB) and systems chemistry (Sections 4, 5). This dual heritage enables WNAI to open a new experimental space:

- unlike hardware-based systems such as electromechanical embodied AI, WNAI enables the investigation of self-organizing cognitive processes beyond mechanical modularity (Section 2.1);
- unlike biologically derived systems, including xenobot-like biohybrids, it allows bottom-up construction from non-living matter, offering finer control over the design, variability, and scalability of cognitive architectures (Section 2.2).

Notably, WNAI is not a chemical/biological variant of neural AI, nor a soft-material implementation of conventional cognition. It articulates a distinct experimental hypothesis: that cognition can emerge from the dynamic closure of reticular chemical systems, where the cognitive and the physical are co-dependent aspects of a single, networked organization. This hypothesis resonates with the enactive tradition (e.g., Di Paolo et al., 2017; Di Paolo and Thompson, 2024) but departs from its biocentric assumptions, extending the possibility of cognitive emergence beyond living organisms (Section 3).

Rather than presupposing the biological as a given, WNAI investigates how cognition may arise in synthetic, prebiotic, or radically non-organic substrates, provided they support the minimal organizational conditions of autonomy and sense-making. On these grounds, WNAI also highlights that a form of embodiment is in principle present in artificial neural network architectures, as they have been originally conceived as analog circuits. Nevertheless, most current implementations of artificial neural networks are on digital hardware; therefore, these are in fact numeric simulations of a physical model. However, notable results have been recently attained with ‘in materia’ network implementations (Milano et al., 2022; Pistolesi et al., 2025). While not physically realized in a robotic or biological body, these architectures exhibit reticular organization and dynamical constraints that can be interpreted as a form of material embodiment. This opens the way for a more integrative view, in which neural and chemical networks are understood as complementary expressions of material–organizational cognition.

This proposal, submitted as a *Hypothesis and Theory* article, addresses three interconnected research questions.

A. What novel embodiment strategies, grounded in the thesis of cognitive networks, can complement existing embodied approaches in the exploration of minimal autonomous artificial cognition?
B. In what ways can SB and chemical systems serve as experimental platforms for constructing and exploring minimal networked cognitive autonomous agents?
C. How can the integration of neural and chemical architectures be realized to advance a plural paradigm for artificial cognition grounded in network-based principles?

To engage with these questions, WNAI advances a dual ambition:

I. To offer a theoretical and methodological framework for developing minimal cognitive agents in synthetic wetware—i.e., chemical substrates with non-equilibrium dynamics and capacity for self-sustained organization;
II. To promote a pluralistic, network-based paradigm in AI that foregrounds organization over substrate while insisting on the constitutive role of materiality in cognition.

By aligning with research on chemical neural networks, synthetic cells, and substrate-sensitive architectures, WNAI aims at providing a testable and generative direction for expanding the scope of artificial cognition beyond silicon and mechanics. This approach is relevant not only for the emerging field of *Wetware Neuromorphic Engineering*, which is central to this Frontiers in Robotics and AI Research Topic—but also for the broader aim of diversifying the epistemological foundations of AI, fostering a landscape in which multiple forms of embodiment, organization, and sense-making can coexist and cross-fertilize.

To situate this proposal within the current research landscape, the next section examines two major trajectories in embodied artificial cognition—embodied AI and xenobotic AI—and outlines how WNAI complements both material and epistemological dimensions of these approaches.

## 2 Positioning WNAI within the field of embodied artificial cognition

The growing attention to embodiment signals a critical shift in artificial intelligence research: from the optimization of disembodied symbol processing or statistical learning systems, toward the construction of cognitive agents whose behavior is shaped by their material organization and environmental interactions. Developments such as the recognition of large language models’ limitations (e.g., LeCun, 2025), advances in biohybrid robotics (e.g., Blackiston et al., 2021), and progress in neuromorphic substrates (e.g., Chen et al., 2024; Halužan Vasle and Moškon, 2024) have underscored that the substrate of cognition is not neutral. The material properties and organizational dynamics of a system play a constitutive role in shaping its cognitive potential.

Within this evolving landscape, two major trajectories have dominated the exploration of embodied artificial cognition: electromechanical approaches (embodied AI) and bio-derived synthetic organisms (xenobotic AI). WNAI complements both, offering a third pathway based on fully synthetic chemical networks. This complementarity is not only material—each approach relying on a distinct substrate—but epistemological, reflecting different assumptions about the nature and genesis of cognition.

### 2.1 Embodied AI: from morphological coupling to network organization

Since the early 1990s, embodied approaches have profoundly influenced the direction of AI research. Grounded in the insight that cognition emerges through the coupling between a system’s organization and its sensorimotor engagement with the environment (Varela et al., 1991), pioneers such as Brooks (1991), Steels (1998), Minoru Asada (e.g., Lund and Asada, 1998) and Rolf Pfeifer (e.g., Pfeifer and Scheier, 1999; Pfeifer and Bongard, 2006) reframed the body not as a constraint but as a generative matrix for cognitive processes. Early strategies leveraged morphological computation and passive dynamics to exploit the physical properties of the body in shaping behaviour, while subsequent developments integrated perception, action, and control in increasingly sophisticated architectures (Damiano and Stano, 2018; Duan et al., 2022).

Over the decades, this trajectory has diversified into multiple lines of work, ranging from reactive agents and behaviour-based robotics to architectures incorporating real-time learning, adaptation, and multimodal perception. Recent research has expanded the material basis of embodied AI, exploring neuromorphic and polymeric substrates, as well as morpho-functional architectures capable of endogenous reconfiguration (e.g., Sandini et al., 2024). These developments have reinforced the view that embodiment is not merely an implementation detail, but a constitutive dimension of cognition, in which the body’s material and organizational features actively shape cognitive potential.

Within this rich tradition, WNAI aligns with embodied AI in its emphasis on the constitutive role of embodiment, while proposing a complementary material strategy: chemical reticulation. Instead of grounding minimal autonomy in physical morphology or sensorimotor loops, WNAI investigates how it can arise at a pre-morphological scale through the organizational closure of self-organizing chemical networks. This perspective resonates with approaches in SB (Damiano and Stano, 2018; 2023) that aim not to replicate existing biological forms, but to construct novel organizational dynamics capable of autonomy and interaction. By focusing on network organization rather than anatomical instantiation, and on chemical self-constitution alongside mechanical integration, WNAI expands the design space of embodied cognition. This complementarity opens possibilities for chemical networks to serve as alternative or hybrid controllers in soft robotics, microrobotics, and other domains where silicon-based architectures may be less effective, thereby enriching the plural landscape of embodied AI research. Building on the embodied AI tradition—from early behaviour-based robotics to current research on morpho-functional architectures—WNAI extends this lineage into the chemical domain, maintaining the focus on material–organizational coupling while shifting the substrate of investigation.

### 2.2 Xenobots and synthetic organisms: a biological route to embodied autonomy

Parallel to developments in electromechanical robotics, recent years have witnessed the emergence of a biologically grounded strategy for exploring artificial cognition. Xenobots—synthetic organisms made of living cells—represent a pioneering class of agents in this domain. Constructed through the reconfiguration of embryonic *Xenopus laevis* tissues, they exemplify a novel form of “wetware embodiment” (Blackiston et al., 2021; Levin, 2021), in which minimal autonomous behaviours—such as locomotion, collective action, regeneration, and environmental memory—emerge from biological morphogenesis rather than from designed control architectures.

Unlike traditional embodied AI, xenobotic AI operates through the intrinsic plasticity and responsiveness of living matter. Xenobots’ behaviour is not the result of pre-programmed loops but of bioelectric and biomechanical processes that self-organize in response to environmental cues. Levin (2021) interprets these capacities as evidence that cognition can be understood as a distributed property of living matter, capable of manifesting in the absence of a nervous system or neural control. These capacities—including agency, persistence, and goal-orientation, described by Joy (2024) as “Stage 1 cognitive traits” — delineate a radically different route to artificial cognition, grounded in developmental biology and tissue-level computation.

While these capabilities highlight the potential of living matter as a substrate for minimal autonomy, the approach is constrained by the morphological and organizational boundaries of biological systems. Xenobots inherit evolutionary legacies that shape their developmental pathways, and their current design relies on top-down tissue engineering rather than bottom-up synthetic construction.

In this landscape as well, WNAI positions itself as a complementary approach. Rather than relying on biological tissues or evolutionary substrates, it explores the possibility of constructing synthetic agents from non-living chemical components capable of self-organization and adaptive behaviour. Whereas xenobots exemplify how minimal cognition can arise within living tissues absent of neural networks, WNAI asks whether similar forms of minimal cognitive autonomy can emerge in non-biological substrates, through the closure and dynamical regulation of chemical networks.

This complementarity is twofold. Materially, WNAI offers a bottom-up synthetic, non-biological alternative to cellular wetware. Epistemologically, it foregrounds design principles that do not inherit from evolutionary or developmental constraints, but emerge from first-principles modelling of autonomy and self-organization. Together, xenobot research and WNAI delineate a broader paradigm shift: one in which embodiment is no longer confined to mechanical or neural substrates, but becomes a matter of reticular organization and dynamic plasticity, realized through diverse forms of material instantiation. In this shift, biological and synthetic strategies can function not as competing paradigms, but as complementary avenues for probing the nature and limits of embodied cognition.

### 2.3 Three complementary strategies for embodied AI

The three approaches outlined above—mechanical, biological, and synthetic chemical—open distinct pathways for the emergence of minimal autonomy, adaptive regulation, and situated behaviour. Table 1 summarizes their defining characteristics, highlighting how each treats the relationship between matter, organization, and cognition in its own terms. This comparative intends to preliminarily clarify the specific contribution of WNAI as a chemically embodied model grounded in reticular self-organization, and to set the stage for the following sections, where we examine how chemical and neural architectures can converge within a shared framework of network-based artificial cognition.

**TABLE 1:** WNAI as a complementary strategy within the landscape of embodied artificial cognition.

Dimension	Embodied AI	Xenobotics	Wetware network-based AI (this work)
Material basis	Electromechanical components	Living biological tissues	Synthetic chemical networks (non-living)
Origin of organization	Engineered modularity	Developmental morphogenesis	Bottom-up chemical self-organization
Artificiality of the system	Designed and built from artificial materials	Assembled from biological cells; partially natural dynamics	Constructed from non-living matter; fully synthetic organization
Autonomy	Programmed control or reactive behavior	Bioelectric and biomechanical responsiveness	Emergent from network closure and regulation
Substrate	Silicon-based, rigid or soft hardware	Cells, tissues from *Xenopus laevis*	Prebiotic/synthetic matter with non-equilibrium dynamics
Cognitive scope	Perception–action loops; embodied control	Minimal behaviors (movement, regeneration, memory)	Adaptive sense-making and related agency
Epistemic contribution	Morphological computation; integration of body and control	Demonstration of cognitive traits in living systems	New experimental platform for substrate-sensitive artificial cognition
Complementarity	Enables integration with physical environments and robotics	Reveals cognitive potential of biological matter	Opens new design space for artificial cognition in synthetic media

## 3 From network cybernetics to pluralist networked AI

In this article, we employ the expression “network cybernetics” to refer to the research program inaugurated by Warren McCulloch and Walter Pitts in 1943. This program aimed to scientifically characterize cognitive processes by formulating hypotheses, expressed in terms of plausible neurophysiological mechanisms, to be systematically tested and refined through the synthetic method–the signature of cybernetic inquiry, encapsulated in the maxim “understanding by building” (Cordeschi, 2002; Damiano and Stano, 2023).

The term network cybernetics emphasizes the central premise of this approach: the then-groundbreaking naturalistic hypothesis that cognitive processes emerge from neural impulses propagating through networked organized brain structures. This was formalized in the McCulloch-Pitts synthetic model presented in the landmark 1943 paper “A Logical Calculus of the Ideas Immanent in Nervous Activity” (McCulloch and Pitts, 1943), where idealized neural networks were implemented using Boolean logical operators configured in specific arrangements.

Our wetware network-based AI (WNAI) research program finds its conceptual roots in this modeling approach, particularly in three often-overlooked aspects that are crucial for rethinking the legacy of the network cybernetics.

### 3.1 First

The central epistemological contribution of the 1943 McCulloch-Pitts paper, which can be recognized in the proposition that the cognitive mind–contrary to the Cartesian *res cogitans*–is not a “substance”, but rather a process: a dynamic phenomenon immanent to, or distributed across, a network of (computational) operations. Indeed, the 1943 McCulloch-Pitts cognitive network model portrays the mind as materially grounded and processually embodied within a reticular chain of operations carried out by elementary components.

*Significance for WNAI* This perspective underpins an understanding of the cognizer as inherently inseparable from its material substrate, being instantiated precisely through the networked operations of interconnected elementary components.

### 3.2 Second

A pluralistic theoretical approach to the development of the cognitive network model, whose explicit articulation can be recognized in McCulloch’s notion of “embodiments of mind” (McCulloch, 1965). Notably, the 1943 paper employed a deterministic perspective, designing networks of ideal neurons to execute specific cognitive functions. This approach, as widely acknowledged by historians of cognitive science and AI, provided relevant conceptual bases for both symbolic computationalist models and later connectionist developments, ultimately contributing to the rise of neural network-based AI technologies–including the engineering-driven advances of deep learning, where networks became powerful function approximators. Yet, importantly, based on their recognition of the oversimplifications embedded in the 1943 model, McCulloch and Pitts presented a markedly different and more biologically plausible framework in their 1947 paper “How We Know Universals” (Pitts and McCulloch, 1947). Here, they depicted the brain not as executing distinct cognitive functions along predetermined neural pathways, but as dynamically generating neuronal networks through self-regulation in response to environmental perturbations. This shift entailed a profound theoretical reorientation: from heteronomy to autonomy; from a passive logical machine receiving exogenous information to a situated biological system actively engaging with its environment; from cognition as problem-solving to cognition as learning; from networks conceived as input-output devices to networks organized through circular causality; and from encoding an independent external reality to enacting meaning through adaptive self-regulation in relation to perceived environmental dynamics.

*Significance for WNAI* This trajectory indicates a critical departure from the classical ways of doing science, suggesting that only a pluralistic integration of approaches can adequately account for the complexity–the material, embodied, and self-organizing dimensions–of cognitive systems.

### 3.3 Third

The second version of the McCulloch-Pitts cognitive network model also contributed to the formation of a distinct research tradition, recognizable as one of the foundational trajectories of embodied cognitive science and AI–particularly the alternative line known as “radical embodiment” (Thompson and Varela, 2001). Here we refer to this trajectory as the *network cybernetics tradition*, which progressively elaborated the 1947 version of the McCulloch-Pitts cognitive network model by emphasizing reticularity as a key feature of cognitive systems. This tradition was grounded in the hypothesis of a deep continuity between life and cognition. More precisely, a “life = cognition” equation, where these terms are understood as two complementary expressions of the defining property of living systems: autonomy, broadly conceived as a form of self-determination involving both self-production and self-regulation. As shown in (Ceruti and Damiano, 2018), the principal architects of this development–Heinz von Foerster, Humberto Maturana, and Francisco Varela, all directly connected to McCulloch through collaborations–produced three influential variants of the McCulloch-Pitts cognitive network model: the biological computer (von Foerster, 1960; von Foerster, 2003), the autopoietic system (Maturana and Varela, 1973; Maturana and Varela, 1980; Varela et al., 1974), and the autonomous system, also referred to as the “conversational unit” or “emergent self” (Varela, 1979; 1991; 1995). In their progression, these models gradually liberated the notion of cognitive network from its original confinement within the brain–more precisely, within neuronal networks. They charted a conceptual trajectory in which the basic cognitive unit, conceived as self-organizing network of operations, was successively identified with whole organisms (von Foerster, 1960; von Foerster, 2003), then with minimal living units or cells (Maturana and Varela, 1973; Maturana and Varela, 1980; Varela et al., 1974), and ultimately with any autonomous entity distinguishable by an observer at a given level of reality (Varela, 1979; Varela, 1991; Varela, 1995). At the same time, they freed the understanding of autonomous network cognition from the notion of computation, developing a constructivist perspective that characterizes it as “sense-making” – or the enactment of a world. In other words: the association of meanings, expressed in terms of self-regulation patterns, to events perceived as perturbations–disturbances in the system’s own dynamic of self-definition.

*Significance for WNAI* At the heart of an autonomous system’s cognition lies the self-regulation of the processes that constitute its network. Autonomous cognitive networks should therefore be understood as systems that, while defining and sustaining their own identity, simultaneously generate (or enact) a world against the background of environmental perturbations.

### 3.4 Modeling perspectives

From a modeling perspective, our programmatic ambition is to advance this tradition along the trajectory opened by Varela’s theory of autonomous systems, by developing wetware systems in which cognition and substrate are intrinsically co-constituted through networked dynamics. As we will show in Section 4 and 5, this is a program deeply rooted in the network cybernetics tradition. It represents a direct filiation of the line of research in SB known as “chemical autopoiesis”, which Varela established together with the chemist Pier Luigi Luisi (e.g., Luisi and Varela, 1989). While recognizing the inherent limits of replicating the full organizational complexity of autopoietic embodiment, we aim to construct *ancestral chemical selves*: minimal self-organizing chemical networks, in which the emergence of an autonomous self, capable of sense-making, coincides with the production of its own substrate.

Recent proposals such as the *Free Energy Principle* (FEP) have also drawn on elements of this lineage—particularly autopoietic biology—to ground formal models of autonomy and adaptive behavior (e.g., Friston, 2010; Kirchhoff et al., 2018; Ororbia and Friston, 2024). While this convergence reflects a broader recognition of the role of self-organization in cognition, it has raised critical questions regarding epistemological alignment. As several authors have noted (e.g., Bitbol and Gallagher, 2018; Di Paolo et al., 2022), despite shared terminology, the inferential structure of FEP may reintroduce representational assumptions that are at odds with the operational understanding of autonomy developed in the autopoietic and enactive traditions. A distinctive feature of WNAI lies in its synthetic and constructionist orientation, aiming to instantiate cognitive autonomy through materially grounded systems rather than through formal inference. This divergence does not indicate incompatibility, but rather exemplifies the plurality of strategies that can emerge from a shared conceptual root. It further reinforces the need for a pluralist epistemology in artificial cognition research—one that embraces different interpretations and instantiations of autonomy.

As anticipated, this modeling orientation not only grounds the development of a novel form of embodied artificial cognition, based on wetware systems that operate fluidly across prebiotic, biological, and ecological realms, marked by minimal invasiveness, intrinsic adaptability, and deep ecological entanglement. It also calls for a pluralistic paradigm of network-based AI, that explicitly acknowledges the genealogical connection between wetware and neural artificial networks, both rooted in the epistemological legacy of McCulloch and Pitts. This shared lineage foregrounds a key assumption: cognition emerges not just from dynamic reticular organization, but from the interplay between that organization and the specific substrate–whether silicon, neuromorphic materials, or chemical media. In other words, the substrate is not a neutral carrier; it actively shapes, constrains, participates in cognitive function, becoming an integral part of how cognition is realized. This cognitive pluralism lays the groundwork for positioning WNAI as a catalyst for cross-fertilization between wetware and neural approaches, opening a dynamic space where distinct forms of artificial cognitive networks can converge, exchange insights, and collectively expand the boundaries of what autonomous cognition can be.

## 4 Defining and operationalizing minimal sense-making: from experimental epistemology towards a chemical program

The transition from the epistemological framework to the chemical program of WNAI requires clarifying how cognition can be recognized and investigated in material systems. Because WNAI proposes a new form of artificial cognition grounded in chemical self-organization, it must specify under what conditions such organization can be said to exhibit sense-making. Without this operational clarification, the framework would remain a theoretical projection, lacking the criteria needed for experimental testing. This section therefore establishes the methodological interface between WNAI’s conceptual foundations and its prospective implementations.

It introduces an operational definition of sense-making, identifies observable features that distinguish minimal cognitive behavior from mere reactivity, and formulates criteria that can guide the design and evaluation of chemical experiments.

By grounding the concept of cognition in measurable forms of self-regulation and structural plasticity, this section prepares the transition from epistemological modeling to experimental realization, defining the conditions under which (minimal) cognition becomes an empirically tractable property of wetware systems.

### 4.1 From chemistry to cognition: operational definition of sense-making

In the WNAI framework, *sense-making* is operationally defined as the emergent capacity of a chemical network to modulate its own structure—understood as evolving sequence of patterns of activity—through self-regulation in response to environmental perturbations, thereby maintaining its organization and identity as a system.

Consistent with autopoietic and enactive approaches, this definition implies that, within WNAI, cognition is not inferred from symbolic representation or neural computation but from *observable adaptive continuity*—from the way a system’s endogenous dynamics selectively adjust to environmental changes through self-regulatory patterns while preserving network closure. In this sense, the transition from chemistry to cognition does not correspond to a categorical leap but an *organizational threshold*: when a chemical network displays autonomous regulation of its own dynamics under varying endogenous conditions, it can be regarded as a *minimal cognitive system*.

### 4.2 Operational criteria and observables of minimal cognition

To make the concept of sense-making empirically tractable, WNAI identifies a set of observables that can indicate the presence of minimal cognitive behavior in chemical systems (Table 2). These criteria distinguish adaptive, self-regulating dynamics from mere reactivity.

**TABLE 2:** Operational criteria for minimal cognition in WNAI systems.

Observable/Metric	Description	Cognitive relevance	Examples of possible chemical correlate
Structural persistence under perturbation	The system maintains its organizational identity while reorganizing its structure (patterns of activity) when exposed to external change	Indicator of autonomy: The network remains organizationally closed despite structural fluctuations	A chemical system that, as a whole, maintains its structural organization and functional identity within the timescale of interest, despite continuous chemical turnover of its components. For example, in the case of autocatalytic networks, even when their dynamics are slowed or accelerated by substituting their substrates with slightly modified structures (replacing the previous ones to perturb the system), autocatalysis persists, illustrating structural persistence under perturbation
Adaptive modulation of structural couplings	Couplings of internal processes change in a way that sustains organizational closure and compensates for environmental variation	Indicator of sense-making: The system interprets perturbations in terms of its own viability conditions	Chemical networks can alter their structure either by generating new nodes—reflecting the potential for autonomous creativity/innovation—or by modifying the parameters that define their connections, such as thermodynamic or kinetic constants, or by the onset of novel causalities. These parameters can be influenced by the network’s internal state (e.g., pH variations) but also by external environmental conditions (e.g., entering in a high osmotic pressure region). The resulting picture is one of multidimensional network plasticity, enabling dynamic modulation of the system’s behavior both internally and in response to environmental interactions
Energy-gradient maintenance	The network sustains a far-from-equilibrium state by redistributing energy flows through structural adjustments	Indicator of self-regulation: Continuous maintenance of the operational boundary	Chemical systems existing out-of-equilibrium need the maintenance of energy gradient, producing entropy in the environment, while maintaining internal order (i.e., their constitutive processes are organized). Without an energy gradient, e.g., energy flux, chemical potential difference, they would relax back to equilibrium. Therefore, two concomitant and necessary mechanisms are required, that is, a mechanism to absorb forms of energy, and a mechanism to degrade them. This is realized by coupling, directly or indirectly (via the network of causal relations) endoergonic and exoergonic processes
‍Plasticity of boundary interactions	The system modulates its permeability or exchange rates with the environment without compromising organizational closure	Indicator of adaptive coupling: Controlled openness that preserves identity	For example, a system enclosed by a membranous boundary may experience increased membrane rigidity due to variations in external conditions—such as the presence of precursors that generate membrane-rigidifying compounds. In response, the membrane autonomously compensates by downregulating the uptake of these specific precursors, as the increased rigidity selectively impairs their binding without inhibiting the binding of others (this behavior exemplifies a negative feedback mechanism)
‍Emergent structural pattern differentiation	New stable patterns of activity arise that enhance the system’s capacity to maintain closure	Indicator of proto-innovation: Structural enrichment compatible with identity maintenance	This may arise through various mechanisms, including autoinduced modifications of rate constants and binding equilibria, altered interaction strengths, and surface-mediated effects. The changes can ultimately trigger the emergence of novel components and catalysts, induce new morphologies, spatial segregations, and gradients of (electro)chemical potentials, as well as alter mesoscopic physical properties—collectively giving rise to distinct emergent patterns

### 4.3 Response and adaptation

If cognition, in line with autopoietic and enactive approaches, is understood as self-regulation that generates meaning—that is, as the stable association of endogenous self-regulatory patterns with exogenous perturbations—then the distinction between response and adaptation can be drawn as follows.

A response corresponds to a transient structural change directly triggered by an external cause; it reflects the immediate impact of a perturbation on the system’s dynamics without endogenous reinterpretation. An adaptation, by contrast, implies a self-consistent restructuring of the system’s own network of reactions aimed at maintaining its operational closure and viability. In this case, the perturbation is not merely reacted to but actively associated with one of the system’s own regulatory patterns, as part of its endogenous dynamics. This adaptive process may take two forms: the selective activation of an existing—already exhibited—self-regulatory pattern within the network’s repertoire, or the emergence of a new, self-consistent pattern that reorganizes coupling between the system and its environment. While the former expresses adaptive flexibility within a stable operational regime, the latter constitutes genuine structural plasticity—a minimal form of learning through which the system enriches its repertoire of viable responses.

Only these latter processes can be meaningfully interpreted as minimal cognitive behavior, since they express the system’s capacity to establish endogenous significance relations between environmental events and its own viability conditions.

### 4.4 Transition towards the chemical program

The operational framework outlined above defines the empirical and conceptual conditions under which cognition can be meaningfully attributed to chemical systems. By specifying the forms of self-regulation and structural plasticity that qualify as minimal sense-making, it delineates the requirements that any experimental implementation of WNAI must satisfy. In this sense, rather than proposing specific architectures, this section has identified the criteria of realization—the thresholds and observables that demarcate cognitive from merely reactive behavior. These methodological foundations now provide the reference frame for the next step: the formulation of chemical architectures, modeling strategies, and experimental pathways capable of embodying and testing the principles established here.

## 5 From operational criteria to chemical architectures: programs and models within the WNAI paradigm

WNAI now moves from its methodological groundings toward the development of a concrete chemical program. While the principles outlined so far define what must count as cognitive in a chemical system, the next step is begin exploring how such conditions might be realized experimentally. The wetware approach thus becomes the material ground where the principles of minimal sense-making can take shape and undergo testing, opening a new field whose contours are only starting to emerge.

### 5.1 Synthetic biology and systems chemistry

The shift proposed by WNAI—to develop forms of self-organizing embodiments—mostly relies on a specfic wetware strategy, namely, the construction of artificial systems in the (bio)chemical domain where the characteristic dynamics of autonomy can be effectively realized, as it happens in living organisms. SB offers rich opportunities for this exploration, provided that in the present context it is understood in its bottom-up or chemical sense. In particular, we consider the diverse methodologies that use chemical components to build life-like systems and networks from scratch.

These bottom-up or chemical approaches complement both the traditional bioengineering branch of SB (Endy, 2005; Andrianantoandro et al., 2006), often directed toward industrial applications (Dhokane et al., 2023; Zheng et al., 2025), and the more recent area of xenobotics. For example, “bottom-up SB”, “chemical SB”, and related frameworks investigate life-like chemical systems following the tradition of origin-of-life research and prebiotic chemistry, intersected with and empowered by the modern perspective called “systems chemistry” (von Kiedrowski et al., 2010; Ruiz-Mirazo et al., 2014; Ashkenasy et al., 2017; Strazewski, 2019). This field focuses on network-based phenomena such as autocatalysis, oscillations, diffusion-reaction coupling, compartmentation, and, more generally, on the emergence of non-linear dynamics in out-of-equilibrium systems. Such dynamics are also linked to the origin of complex biological patterns—embryonic development, organ formation, or pigmentation—as insightfully conceived by Turing (1952).

The WNAI program we advance to investigate minimal cognitive systems does not coincide conceptually with conventional SB bioengineering, although it may share with it some methodological aspects. Most SB design strategies adopt a computationalist perspective, translating algorithmic reasoning into the wetware domain (for instance, genetic circuits operating as logic gates, Siuti et al., 2013). While productive, this approach remains close to the line inaugurated by McCulloch and Pitts—the construction of neural-like networks performing predefined cognitive functions. In contrast, WNAI explores chemical systems not as devices executing designed operations, but as autonomous self-regulating networks capable of maintaining their organization and generating adaptive behavior. Operationally, WNAI also differs from xenobot research: it does not involve complex living organisms but shares an emergentist and systemic orientation, emphasizing the distributed origin of cognitive capacities.

The role of higher-level organization—typical of developing tissues—is considered a later stage of investigation, to be addressed once transitions from single-cell to proto-tissue organization become feasible. Recent studies on assemblies of cell-like systems (synthetic cells) already point in this direction and represent one of the most promising frontiers of wetware research (e.g., Liu et al., 2022; Valente et al., 2024; Zhang et al., 2024). Reticular and reciprocal system-level interactions–potentially spanning multiple scales–will be central to analyzing these systems within the WNAI framework.

In this perspective, constructing bio-chemical systems, even when similar to standard SB devices, is not an end in itself, but a means to explore network processes that sustain autonomy and autopoiesis. SB plays a pragmatic role, providing techniques and methods for chemical implementation, while WNAI supplies the conceptual interpretation of the resulting wetware models. In the following, we outline the principles guiding the development of WNAI models, their realization, and their potential areas of application. Expressions such as “chemical networks” or “chemical systems” are used interchangeably to refer to the wetware artificial systems targeted by the WNAI paradigm. The issue of self-bounding—a central notion in autopoiesis—is treated here in generalized terms as self-individuation, whose specific realization depends on the domain in which the chemical network processes are defined. We do not provide detailed examples but maintain a general scope, ensuring that the guiding lines of the WNAI paradigm remain clear and conceptually consistent.

### 5.2 Five guiding concepts for design

A generic theoretical model can be built around key conceptual points that summarize the pioneering approaches to self-organization, as introduced above (e.g., Damiano, 2012). In particular, we can identify five concepts that will function as guiding principles for the variety of research programs contributing to the WNAI.

The first concept is that of “organization”, referring to the structured relationships among components that form a self-organizing system. These relationships, which have qualitative and quantitative features, generate an integrated whole with interacting parts. The focus is on the relationships between the parts and between the processes occurring within the system, rather than on the parts or on the processes themselves. There is, then, the concept of “emergence”, which highlights how self-organizing systems—through their specific dynamics–develop novel, irreducible properties at higher levels, where parts and the whole interact dynamically, shaping the system’s overall behavior. “Autonomy”, on the other hand, describes the system’s ability to self-regulate and determine its own structure, maintaining a degree of independence while responding to external influences. The system reacts to external cues in a manner that is constrained from within, from its ongoing processes, restraining the range of possible variations to those compatible with its own existence-assuring processes. “Co-evolution” explains the changes occurring in the structure of system and in the structure of its environment, both being network-like ones. The internal and interfacial relationships between parts and processes adjust dynamically on both sides, leading to mutually compatible behaviors. Co-evolution can be interpreted both in the long and short timescale. The long timescale corresponds to evolutive or phylogenetic time, while the short one refers to here-and-now adaptive processes. The environment, in other words, does not play an “instructive”, but a “co-constructive” role in determining the pathway of the system. Finally, and crucially, the concept of “closure” defines a self-organizing system as a closed network of relationships among its interconnected components, reinforcing the view of the system as a coherent unity. In the formulation proposed by Varela, (1979), this concept generalizes Piaget’s earlier notion of closure (Piaget, 1967) and captures the circular interdependence of processes—and thus of the components’ transformations—that characterizes self- organizing systems (e.g., Ceruti and Damiano, 2018). Importantly, this notion can also be extended to conceptualize the co-evolutionary dynamics between a given self-organizing system and other systems (for example, the environment, as mentioned, or one or more distinct autonomous systems).

### 5.3 Chemical networks and their environment

The core idea is that chemical networks, specifically in the form of cell-like systems or “synthetic cells” (“artificial cells”, “protocells”) (Oberholzer et al., 1995; Luisi, 2002), offer a novel pathway for exploring wetware systems, operating on a peculiar form of chemically embodied AI (the WNAI one), that more accurately model minimal life and minimal cognition–two complementary aspects of autonomy in living beings, according to the autopoiesis theory.

**FIGURE 1:**
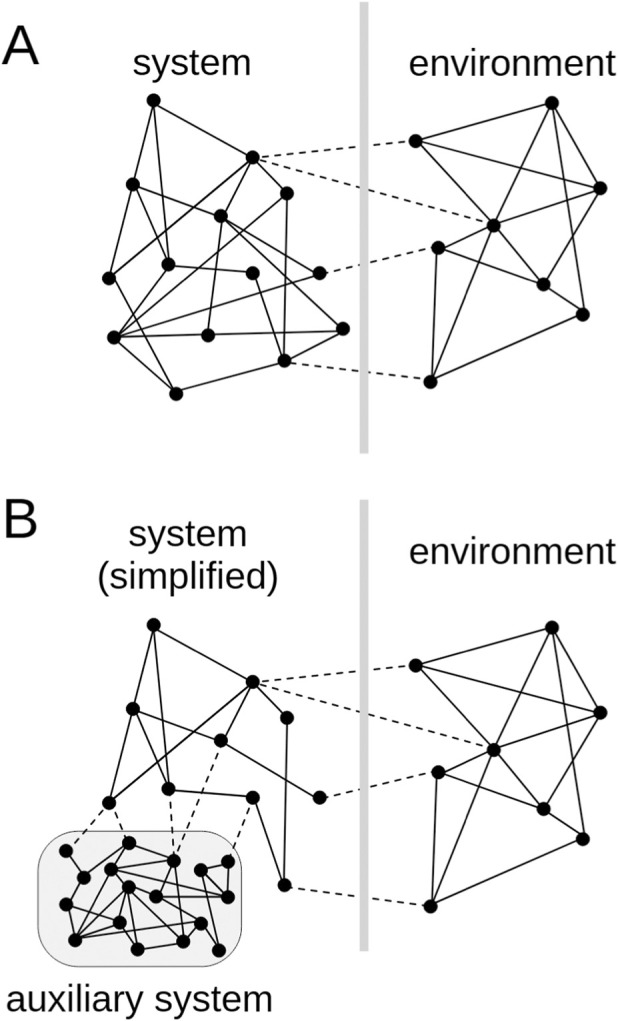
**(A)** Schematic drawing of two networks (the “system” and the “environment” in reciprocal interaction. Inspired by (Ashby, 1960), p. 70. **(B)** To facilitate experimental approaches, some necessary, yet not dynamics-determining processes, can be offloaded to an auxiliary network, which constitutes a sort of inner reservoir for pseudo-stationary exchanges. The auxiliary network should not contribute, or contribute only indirectly, to the autonomous and self-regulating mechanisms of interest.

This approach is promising because chemically embodied AI, or “chemical AI” (Gentili, 2013; Kuzuya et al., 2023) leverages mechanisms that remain fundamentally inaccessible to traditional hardware and software artifacts. Rather than pursing a virtual simulation or mechanical imitation of life-like behavior, wetware models emulate living systems because they populate the same physico-chemical space of existence. To achieve this, the construction of microscopic cell-like systems represents a viable yet challenging approach to embody those reticular dynamical processes that are fundamental to generate an autopoietic system. Cleary, the initial steps require to fix the target to unicellular or paucicellular constructs only, matching the current technological capabilities. Despite their extreme simplicity, these cases remain relevant within the framework of the continuity principle, which holds that every living system—even the most elementary—is inherently equipped with the fundamental characteristics of life, including minimal forms of cognition. In fact, it is evident that all forms of life, in order to survive, have learned about their environment, and navigate reality skillfully, not by using logical reasoning but by other more fundamental modes. The chemical systems we target, indeed, can be described as self-bounded dynamical networks engaging bidirectional interactions with another system (a dynamic environment, also seen as a network of processes), as sketched in Figure 1A. A reciprocal interplay between the network of interest and its surroundings take place, and this fundamental process, a relational one, becomes the key concept for designing artificial chemical AI systems. The strong remark on forms of intelligence that are embodied (chemical networks) and situated (facing an environment with perturbing ability), is a hallmark of our program.

This approach offers a fundamental non-computationalist pathway (rather, a constructivist one) to model minimal cognition. It complements, in the field of bottom-up synthetic cell research, more common approaches generally aimed at reconstructing mechanisms for self-reproduction (e.g., synthetic cell growth-and-division). While the endeavor we propose presents relevant challenges, the minimal complexity of these cell-like systems may help to mitigate some of the difficulties. The potential rewards of these efforts may unlock an unprecedented exploration of foundational forms of artificial intelligence, particularly given that autonomous systems have been described as possessing mind-like characteristics (Varela, 1979). This perspective will potentially offer valuable insights for cognitive science and pave the way for a development of a novel framework to guide, in near future, disruptive new biotechnological tools. Such a design space, while distinct from conventional robotics, addresses critical limitations in scalability, adaptability, and substrate-dependence that affect current embodied AI systems.

The crucial aspects inherent to the chemical domain that will play a decisive role for developing chemical autopoietic AI are as follows.

i. Embody AI in chemical networks. Unlike man-made electro-mechanical machines, which are designed with a strict hardware/software separation, chemical networks can inherently embody forms of AI. In these systems, the components function simultaneously as hardware and software, eliminating the distinction found in traditional computational architectures.
ii. Blur the operator-operand distinction. The conventional separation between operators (computational elements such as enzymes) and operands (substrates) can disappear, generally speaking, in chemical networks. This flexibility introduces circular causality and the blending of these categories.
iii. Exploit the plasticity of transformation rules. In chemical space, transformation rules cannot be predefined. Instead, networks exhibit intrinsic plasticity. The variations in the copy number of chemical species directly influence association constants and reaction rates (therefore, the network structure and its dynamics). Shifts in species concentration can enable new reaction pathways, potentially leading to the emergence of novel compounds and molecular complexes not originally present in the system. The complexes, in particular, might display properties not present in their separated constituents. Network parameters—such as rate constants and association constants—exhibit physical dependencies, for example, on temperature (which, in turn, can vary in context dependent manner, e.g., depending on energy fluxes in the network). Ionic and ionizable compounds (produced or consumed by network processes), through their associated ions and ion release mechanisms, respectively, influence electrochemical potentials, drive current generation, and, in the presence of semi-permeable membranes, produce active transmembrane voltages. In turn, these phenomena affect the network dynamics closing a loop of circular causality.
iv. Enable dynamic reconfiguration and emergent creativity. As a further consequence of (iii), the dimensions of the phase space of a chemical network can change (in particular, it can increase), enabling “creativity” to emerge in unique manner, with novel and unpredictable structures and functions arising. When environmental conditions change—whether at a global or local level—chemical networks dynamically reconfigure their structure in response, but they can also start a genuinely new explorative path. The logic behind these processes of meaning generation has been described by Henri Atlan as the “complexity from noise” principle (Atlan, 1987).
v. Facilitate energy conversion across scales. At the nanoscale, energy conversion between various forms—such as thermal, mechanical, chemical, and electrical—occurs with remarkable efficiency. This is primarily because these energy modalities possess comparable magnitudes at such small scales, facilitating their interplay and transformation (Phillips and Quake, 2006). Unlike in macroscopic systems, where energy domains often differ vastly in scale and coupling mechanisms are less direct, nanoscale systems inherently support cross-domain interactions, enabling sophisticated functionalities in nanotechnology and molecular machines.

Based on these considerations, we believe that the WNAI program will contribute to move beyond the mere replication of conventional computational processes in wetware, disclosing more fundamental forms of intelligence, metabolic/regulatory, vegetative one. These are abilities (or “dexterities”, Guidoni, 2018) that living systems have at all scales. The WNAI program has potential to identify minimal chemical networks equipped with self-regulation and explorative mechanisms, enabling them to adapt to external and internal perturbations. Importantly, these dynamics can be interpreted as systems that become able to autonomously generate meaning—a process known as sense-making–through the generative operations of adaptive self-regulation that we can aptly term “minimal cognition”.

### 5.4 Possible experimental implementations

Looking now at experimental implementation, full-fledged autopoietic chemical AI should, by definition, involve autopoietic systems. However, constructing from scratch such systems in the chemical domain presents significant practical challenges. As a first step of the possible roadmap to this ambitious goal, we propose to deal with autonomous rather than with autopoietic systems. Instead of focusing on a set of metabolic transformations—namely, the cyclic production and decomposition of the network’s chemical components, a necessary condition for autopoiesis—our approach will initially target processes that constitute an operationally closed network of causal relations and are often capable of adaptive, self-regulative behavior. In other words, we propose to focus on systems composed of interdependent processes, topologically arranged in circular or reticular fashion, that function recursively, and that can be recognized as a unified entity within their operational domain (the Varelian definition of autonomy, 1979). The study of autonomous wetware systems with a particular focus on their adaptive and self-regulative responses to perturbative environments offers a promising path toward synthetic approaches to sense-making and generation of meaning, i.e., on synthetic or artificial cognition. This approach aims to develop embodied forms of AI deeply inspired by living systems—not by embedding predesigned algorithms or computational devices into chemical substrates (a strategy encompassed in other forms of “chemical AI”, such as chemical logic gates, genetic circuits, and similar ones), but by exploring whether, and to what extent, biological organization can spontaneously emerge in artificial chemical systems.

The concrete implementation, from scratch, of autonomous chemical systems will be important products of the WNAI program. From a topological viewpoint, closed circular/reticular nets of reciprocally entailing processes are necessary. As Morowitz has put it, circularity is a requirement for out-of-equilibrium, dissipative steady state systems (Morowitz, 1979; see also Plasson and Jullien, 2025). Then, there is the issue of self-bounding, or, more generally speaking, of self-identification in a certain domain. The domain can be interpreted physically (as the case of cell-like systems) or functionally (e.g., as in the case of process sets which operate in the same “space”, e.g., the space of reciprocal transcriptional regulation, as it happens in repressilator dynamics (Elowitz and Leibler, 2000)). To bypass the high kinetic barriers of covalent chemical transformations, which can hinder the onset of dynamical chemical networks, two main strategies appear feasible: the use of catalysts (and, specifically, enzymes and alike), or conceiving dynamical systems based on easy-to-form and easy-to-break covalent or non-covalent bonds. The first strategy can rely on bottom-up or reconstitution SB approaches and transcription-translation systems, or on organocatalysis (Gorlero et al., 2009; Wieczorek et al., 2017; Rout et al., 2025), while the second on currently explored systems chemistry methodologies based on disulfide bonds, imine bonds, coordination bonds, and alike. These bonds exemplify dynamic covalent or coordination linkages whose reversibility and responsiveness make them particularly well suited for constructing adaptive chemical networks. Their ability to sustain self-regulating interactions and to reorganize under changing conditions highlights their relevance as model systems for exploring life-like behaviors and emergent properties in systems chemistry.

Previous reports provide also additional hints for fertile directions in self-bounding systems to look at. It should be recalled, for instance, that pioneer attempts to build autopoietic systems have been reported by Pier Luigi Luisi by using microcompartments such as micelles, reverse micelles, and vesicles (Bachmann et al., 1990; 1991; Walde et al., 1994; Blochliger et al., 1998). The balance of anabolic and catabolic processes can lead to a form of homeostasis (Zepik et al., 2001; Post and Fletcher, 2020; Fracassi et al., 2025; Jha et al., 2025). Coacervates are also interesting microcompartments which have shown many responsive behavior (Gobbo, 2020; Gao et al., 2021; Yin et al., 2023), as well as oil droplets do (Hanczyc and Ikegami, 2010; Parilla Gutierrez et al., 2014; Čejková et al., 2017). The WNAI program is therefore a sort of call to action, for systems chemists, to face the new and highly ambitious plans referred to embodied forms of AI in chemical networks (Gentili and Stano, 2023b; 2024).

To be more specific, the classic model proposed by Luisi and Varela (1989), and experimentally validated by Bachmann et al. (1990), serves as a minimal example of WNAI implementations. The system consists of 50 mM sodium octanoate as surfactant and aqueous LiOH, in 9:l (v/v) isooctane/l-octanol. Physically, the aqueous LiOH solution is dispersed in billions of tiny droplets surrounded by sodium octanoate/1-octanol, i.e., reverse micelles (diameter ca. 5 nm). The alcohol (1-octanol) actually is a cosurfactant, essential for constituting stable reverse micelles. Octyl octanoate is added, it binds to the micelle interface, and transformed into octanoate plus 1-octanol by reacting with LiOH (saponification reaction). This generate more micelle-forming molecules, while the amount of aqueous solution remains constant, decreasing the water-to-surfactant ratio (*w*_0_), ultimately leading to reverse micelle destabilization and division. The process starts again, repeatedly, until depletion of LiOH and/or octyl octanoate.

**FIGURE 2:**
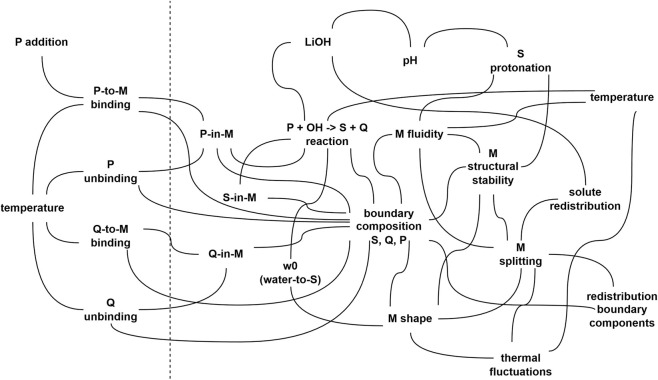
An illustrative version of the general scheme shown in Figure 1A. The network represents a possible set of interconnected variables and processes that can be identified in the autopoietic reverse micelles (Luisi and Varela, 1989; Bachmann et al., 1990), which can serve as a minimal example of WNAI implementations. Variables and processes on the left of the dashed line refer to the environment, while the others refer to the system. Events occurring at the molecular and supramolecular domains are intertwined: the propensities and the rates of processes are *per se* not constant, but depends on the value of certain variables, which in turn depends on the ongoing processes, possibly on a different scale. The composition of the system, e.g., of the reverse micelle amphiphilic boundary, determines the exact value of free energy, ultimately affecting physical as well as chemical processes occurring on the boundary region. Gradients of intensive variables such as chemical potential or surface tension and their conjugated flows are also related. A detailed description of how the system works, in summary, is given in the text (Section 5.4). In this network, M: micelle; P: precursor (octyl octanoate); S: micelle-forming amphiphilic compound (octanoate); Q: byproduct of the P-to-S transformation (1-octanol), also participating to the boundary and present in outer environment, together with isooctane; LiOH: lithium hydroxide (the inner reactant); *w*_0_: [water]/[S] ratio, a major determinant of micellar structure/stability (but it is also context-dependent, i.e., micelles formed in two different solvents, even characterized by the same *w*_0_, are not necessarily similar). It should also be noted that the extremely small size of these systems is expected to introduce stochastic behavior across all processes (e.g., “redistribution of solutes”, “redistribution of boundary components”). The network has been drawn with illustrative purposes, and it is not supposed to be exhaustive of all possible causal connections (e.g., sodium/lithium competition as counterions has not been included). Another illustrative reaction network, based on a simplified model of protein-synthesizing artificial cells, can be found in Gentili and Stano (2023a).

The processes that generate the observed behavior are circularly related by a series of entailments. Indeed, the systems dynamics proceed autonomously without need of external control. The causal cycle can be described as it follows: the precursor octyl octanoate binds to reverse micelles, following a thermodynamically driven process of adsorption; it reacts with LiOH guided by the favourable reaction free energy for a saponification; the products remains at the micelle interface, contributing to vary either the interfacial composition, the interfacial physical features, and the local *w*_0_; the micelle becomes physically unstable and splits into daughter micelles; the reduced molar fraction of octyl octanoate in daughter micelles drives the binding of fresh octyl octanoate; and the cycle re-starts (actually, it is autocatalytic). A representation of the above-mentioned system in form of a network (similar to Figure 1A) is presented in Figure 2, where an extended set of variables and processes has been included.

In order to explore the cognitive features of this system, however, it should be subjected to perturbations and verify whether or not it is able to provide an adaptive, self-regulative response, while keeping its own organization, i.e., by maintaining itself in the class of systems it belongs to (that is, in the class of systems that behave as described above, capable of performing the network of above-specified concatenated operations). If this is the case, the way the system self-regulates will be the sense-making process (generation of meaning, cognition), while all interactions that leave the system unperturbed make no sense for the system. Similarly, interactions that the system cannot compensate (in order to maintain its organization, that coincides also with its existence) do not belong to its cognitive domain. Cognition (knowing), according to this constructivistic, autopoietic perspective, is a form of behaving (doing).

Examples of perturbations potentially perceived by the autonomous dynamics of self-reproducing reverse micelles are expected to arise within the *chemical space* of potential precursors, solvents, reactants, and related components, as well as in the *physical and temporal space* of physico-chemical micelle-environment interactions. Preliminary thoughts about how computer simulations and experiments could be carried out have been briefly outlined elsewhere (Stano, 2022a; Stano, 2023). Structural modifications—such as shortening or lengthening ester carbon chains—can induce changes in the thermodynamic and kinetic parameters of the processes mentioned above, leading to qualitative and/or quantitative alterations in the network architecture. Similarly, if the precursors are delivered as discrete pulses rather than continuously, both the time intervals between pulses and the concentration of precursors become experimental variables amenable to perturbation. These parameters can be adjusted (and later perturbed) to investigate whether and to what extent the system can tolerate noise and literally incorporate self-regulatory patterns–where available. The system’s ability to persist within the transformation space, along with the emergence of self—regulatory behaviors driven by specific molecular interactions, can be validated experimentally. In these cases, the meanings produced by the system are understandably very elementary and should ideally be interpreted through the lens of adaptation rather than permanent learning. Nonetheless, adaptation itself represents a compelling dynamic, especially when contrasted with mere automatic responses. Adaptation should be inferred from deviations in the system’s expected behavior—ideally forming part of a compensatory process—with the caveat that the mechanism should be functional to the continuation of the overall dynamics, that is, the autopoietic growth-and-division mentioned above.

As previously noted, leveraging conventional bottom-up SB tools—especially those based on biomolecular systems involved in cellular regulation and control—also offers a promising pathway toward achieving autonomy. Reconstructed SB networks are paradoxically both simpler and more complex than other chemical networks. On one hand, they are simpler because each transformation is typically governed by a specific protein, which greatly facilitates rational design. On the other hand, their reliance on macromolecular components introduces significant complexity. For instance, establishing a fully self-producing network is difficult, as processes like protein synthesis require complex ribosomal-tRNA mechanisms, hundreds of molecular components, and encoded genetic information.

Given that the focus can be shifted, at least initially, from autopoiesis to adaptive behavior and self-regulation, the construction of autonomous wetware systems could be designed by delegating otherwise important processes to an “auxiliary network” (Figure 1B). This is especially feasible for systems designed by a bottom-up SB. The processes that constitute the auxiliary network should reside in an orthogonal domain (with respect to the adaptive or self-regulative processes of the autonomous network), with the advantage that the auxiliary network can be complexified at will, to match practical requirements. By auxiliary network we mean a system whose sole function is to maintain the network of adaptive and self-regulative processes of interest in the required out-of-equilibrium condition, so that the self-regulative processes could take place without the burden of a concomitant complete self-production of components and precursors. This helper network constitutes a sort of stationary (caveat: for all practical purposes) “inner” environment that assists the network of interest by supplying building matter, energy-rich compounds, or regular signaling cues that excite or inhibit the network of interest, the one under scrutiny for autonomous behavior. In a certain sense, it plays a similar role of what a thermal reservoir is for heat engines: it allows the functioning of the engine, being considered as reliable and stable in a certain timeframe.

The auxiliary network could even share the same physical space of the network of interest but should not operate in the same domain of the self-regulatory processes of interest. The autonomous network, designed to model a target cognitive phenomenon, operates independently from the auxiliary network while involved in its own recursive processes, while the auxiliary support is not engaged in the recursive entailment processes of interest. We are aware that this pragmatic strategy contrasts conceptually with the notion of unity and integration that is implicit in any system-level approach, as the WNAI is, but it should be seen as a necessary compromise for enabling incremental progress toward a more holistic design.

An easy-to-describe example of auxiliary network comes from artificial biochemical networks reconstituted in systems like synthetic cells according to a bottom-up SB approach. Consider feed-forward or, as a best example, closed-loop (recurrent) “chemical neural networks” (Hellingwerf et al., 1995), such as signaling networks or gene regulatory networks like those described in previous proposals (Gentili and Stano, 2022; Stano et al., 2022b; Braccini et al., 2023). These networks operate by generating activation and deactivation of its own components, a form of gene expression regulation. The processes (α, β, γ, …) constitute a space in which the system dynamics resides, the space of regulatory processes. If the network is autonomous (operationally closed), the set of processes completely describe the network dynamics via a set of recursive causations or entailments (α causes β, β causes γ, … etc., in a reticular topology). In simulations, the dynamics of the network of interest and of the environmental perturbations can be investigated without the need of any auxiliary network. In contrary, the wetware implementation of such networks generally requires the inclusion of other chemicals that give rise to additional processes, e.g., providing energy and specific substrates, allowing gene transcription and translation, acting as buffer/stabilizers of metabolite concentration ratios, eliminating byproducts with potential inhibiting effects, and so on. The processes carried out by these auxiliary reactions, do not reside, generally, in the same space (domain) of the processes occurring in the network of interest, i.e., for example, they would be recognized just as metabolic processes. While, actually, they should be consider together with the network of interest (conceiving all processes as a whole, and exploring the behavior of a large complex network), it can be convenient to focus on the network of interest only, especially if the auxiliary processes are, for all practical purpose, non-interfering (orthogonal) with the former. If the separation (in terms of domain, stoichiometric balance, reaction rates, etc.) is permitted, the strategy outlined in Figure 1B enables a more efficient investigation and prototyping of cognitive systems in line with the WNAI principles. In other words, this design should be considered as a shortcut to facilitate practical implementations of autonomous/cognitive systems, but cannot be considered the ultimate design. In fact, the auxiliary system could inadvertently constrain the core network dynamics and the entailment relations (depriving the core network of autonomy), as well as the network plasticity (affecting adaptive or learning behavior).

Within this context it is also useful to recall a comment by Dennis Bray, who highlighted the similarity between the mathematics developed to describe neural networks and the one suited to describing chemical networks (Bray, 2009). Recurrent chemical neural networks have been identified as valuable targets in the context of autonomous wetware agents (Braccini et al., 2023). Artificial recurrent neural networks can achieve in principle the highest level of computational capability among artificial network models; when properly designed and trained, they have shown to be effective in accomplishing tasks that require memory, e.g., prediction of time series. However, their space of possibilities is bounded by their structure: neurons, links and functions. Conversely, wetware recurrent networks are characterized by further dimensions of capabilities that go beyond the computational power of hardware artificial networks. The reason is that wetware networks are inherently open: new chemical species and new reactions can arise in time enabling these systems to evolve in an open-ended context, exploring their adjacent possible (Kauffman, 2016) and extending their current space of possibilities.

The significance of the future experimental approaches in application scenarios is introduced in Section 6 and Supplementary Appendix A. While in some of the described applications a computationalist (input-output; stimulus-response) framework would be considered sufficient for engineering chemical systems with satisfactory performance, we claim that by embracing a constructivist perspective, the one implicit in the WNAI proposal, offer a broader and potentially more rewarding perspective. The WNAI approach focuses, indeed, on the emergence of functions or behaviors as adaptive responses, conceptualizing systems as *complex adaptive systems*. This shift in viewpoint is particularly relevant for more ambitious goals, such as tissue engineering and regenerative medicine, where codependent relationships form among the members of collections or aggregates, and new constraints emerge at a higher organizational level. These constraints, in turn, give rise to behaviors that are observable at that higher level–behaviors that not only depend on the dynamics of individual components, but that are imposed downward on them by the superstructure they compose. A distributed, system-level codetermination emerges as a new form of self-organization, echoing the sets of relationships between cells, molecules, and the intracellular network they originate. It should be added that terms like “response” and “adaptation” are frequently used—and at times conflated—in recent literature to describe the behaviors of such systems. While “response” typically denotes pre-programmed stimulus-response (input-output) dynamics, we suggest that “adaptation” should be reserved for cases involving unprogrammed, self-modifying behavior characterized by internal rule changes, such as learning. However, this distinction can become increasingly slippery when examined within the framework of chemical networks. Our ongoing research is specifically focused on unpacking the nuances of meaning embedded in this classification, and the findings will be detailed in a forthcoming publication.

### 5.5 Challenges and limitations to be considered

From a technological standpoint, specific limitations may arise related to stability, timescales, and—generally speaking—noise. The stability of chemical networks must be considered at both the molecular and supramolecular levels. One of the most fascinating yet challenging perspectives on wetware cognitive chemical systems is that the phenomena of interest span multiple scales, representing a case of “multiscale” chemistry. At the molecular scale, reaction stability ultimately depends on the availability of reactants, the removal of products, and the potential onset of undesired side interactions. Maintaining a continuous chemical flow is essential in non-equilibrium systems; otherwise, the network structure may collapse as the net flux of matter between network nodes diminishes. Consequently, the design must incorporate mechanisms that fulfill this fundamental requirement of chemical reactivity. However, as we have repeatedly emphasized, the ability to modulate these flows—i.e., the system’s plasticity—is also crucial for achieving adaptivity.

The importance of the timescale on which network dynamics operate is another crucial factor in the successful design of cognitive chemical systems. While such dynamics could, in principle, span a range of timescales, it is evident that self-regulatory mechanisms must occur on timescales comparable to those of the perturbations. Otherwise, the system may collapse before adaptation can take place—not due to the absence of adaptive mechanisms, but because they operate on a slower timescale. A related discussion on self-regulative processes in cognitive system must be mentioned (Bich and Moreno, 2016; Bich et al., 2016). These authors have commented on regulatory subsystems that, in organisms, constitute a set of processes dynamically decoupled from the dynamics of their constitutive regime–the regulated subsystem (meaning that the constitutive, or regulated, and the regulating subsystems work at distinct intrinsic rates–see also Pattee, 1977).

Noise—often regarded as a hindrance to chemical computation, particularly because of its association with the conventional role of noise in communication theory and its correlation with the transposition of Boolean logic into the chemical domain—assumes a different role within the WNAI framework. As highlighted by Henry Atlan, noise can have beneficial effects, contributing to the emergence of complexity (and meaning) in systems that possess sufficient redundancy (Atlan, 1987; Varela, 1988; Roli et al., 2024). Using concepts from information theory, Atlan demonstrated that while noise typically acts as a disturbance in a communication channel, its impact changes when viewed from a higher level that encompasses both source and destination as components of a broader, multi-scale organized system. Adopting this systemic perspective, noise enriches the diversity of intra-system causal pathways, ultimately fostering more intricate relationships among system components and enabling the emergence of new meanings. The meaning that noise or perturbations may carry for the system cannot be predefined *a priori*; it depends entirely on the system’s capacity to accommodate certain stimuli. In other words, the meaning will be co-determined by the system structural organization and by the manner it adapts to the disturbances.

With respect to scalability, a vision for increasing complexity in chemical networks embraces a multi-scale perspective. In this article, we have primarily focused on the general concept of autonomous chemical networks, which may naturally evoke analogies with intracellular biochemical systems. However, processes involving boundaries can be readily incorporated. Scaling up the organization implies a transition from individual autonomous, cell-like systems to collective assemblies (Carrara et al., 2012). These systems are now commonly referred to as tissue-like assemblies, or prototissues, and represent a novel level of organizational complexity (Liu et al., 2022; Valente et al., 2024; Zhang et al., 2024). The shift has two major implications. First, the prototissue imposes constraints on the processes permitted at the lower level–constraints that are essential for the prototissue’s very existence. For instance, if cell-like systems aggregate via adhesive molecules produced internally and exported to their surfaces, then perturbations that (i) significantly alter the quantity of these molecules, or (ii) disrupt export mechanisms, or (iii) introduce inhibitors of molecular recognition, must be constrained within a narrow range of variability. Second, the prototissue introduces new domains of interaction that are absent at the single-cell level. For example, mechanical deformations and release/uptake of certain chemical species can act as perturbations generated from an individual within the assembly to another. Even the position of an individual in an assembly can generate differentiation at a very basic level (e.g., being at the center or at the periphery is itself a source of diversity and can determine different optimal behavioral, that is, metabolic patterns). Scaling up the layers of complexity enables the system to perform increasingly sophisticated operations because these operations resides in domains different than the basic metabolic ones. An example can be the search for optimal metabolic rates depending on the position of individuals in the assembly. These can be interpreted as forms of information processing or, in our perspective, as the emergence of novel meanings. Regardless of the epistemological lens, what matters is that the scope of problem-solving and decision-making expands—consistent with the concept of cognitive domains, as emphasized also by others (e.g., McMillen and Levin, 2024).

### 5.6 A tentative roadmap

The WNAI program outlines a tentative roadmap for the realization of wetware-based artificial cognitive agents. This roadmap identifies three complementary directions that, while technically challenging, are partially grounded in current research and represent viable steps for exploration and future implementation.

1. Focusing on the construction of artificial chemical networks and importance of compartmentalization. The development of microscopic, cell-like systems—often referred to as synthetic cells, artificial cells, or protocells—could provide a concrete experimental platform. These systems are *envisioned to embody* reticular dynamical processes that may be necessary for the generation of autopoietic/autonomous behavior. Even in their simplest forms, they *might be able to* express basic forms of cognition, understood here as minimal sense-making emerging from structural coupling with a dynamic environment.
2. Generation of emergent dynamics through wetware networks. Rather than reproducing biological mechanisms as an intrinsic goal (for constructing a cell from scratch), this approach *would aim* to realize organizational principles of living systems in chemical networks. Instead of casting pre-formed algorithms in the chemical substrate, these networks *could take shapes that* dissolve traditional separations between operator and operand, hardware and software, favouring an integral/distributed vision. This should enable intrinsic plasticity, circular causality, and adaptivity. As a workable experimental strategy, the utilization of *auxiliary networks* has been proposed (in particular, in bottom-up SB approaches). In some cases, necessary but not-functional roles may be externalized to auxiliary networks not operating within the operational domain of the system of interest. This strategy facilitates experimental design without compromising the autonomous dynamics of the core wetware agent. Figure 1B illustrates such an arrangement, where auxiliary components support—without replacing—the reticular organization of the system under investigation.
3. Exploiting situatedness and system-environment interactions. This is, possibly, the least investigated aspect so far. To date, many researchers have focused the attention of how systems (e.g., synthetic cells) behave in a given environment, supposed to be fixed. Clearly, this stems from the necessity of developing molecular machinery in a precise and function-oriented manner. However, when cognition takes center stage, environmental variations, divergences, and perturbations emerge not as noise, but as essential co-constructive features of the cognitive processes. Under the new perspective, systems and their environments should always be considered together, as a super-system, and the resulting behavior will be conceived as a relational property of the two (Figure 1A).
4. Identifying observables and metrics to verify that the systems under scrutiny is cognitive. The generation of meaning (or sense-making), that is, the realization of cognitive capabilities, occurs in the domain of the operations the autonomous system perform to compensate the perturbations it receives from outside or from inside—while maintaining its identity as an element of the class of systems it belongs to. Therefore, in order to observe its occurrence, there is a need of an accurate scrutiny of system dynamics, to decifer the way it is organized and the way in which it diverges from its pre-existing form. Indeed, the way the system compensate perturbations is the way it knows them. By organization we intend the topological and nomological description of the network-like set of processes that determine its existence as an out-of-equilibrium chemical system, and the way it interact with environmental variables. It follows that the observables, in these systems, are those variables that concur to its exact chemical description, i.e., the measurements of the concentration-vs-time profiles of all species, complemented with morphological analysis (e.g., the shape in the case of synthetic cells), the monitoring of electric potentials, the macromolecular conformations (when relevant), etc. The task is not trivial. Reasons like low concentrations, intrinsically unfavored signal-to-noise ratio, availability of real-time revealing techniques, absence of analyte-specific signals and separability of signals, are among the causes of these difficulties. However, it is often possible to characterize systems by monitoring few particularly relevant variables whose changes coarse-grain, or can be conceived as proxies for hidden and intricate variations of many other variables that they cannot be measured at the desired precision, and/or without destroying the system. The challenge for the experimenter, therefore, is the identification of suitable variables to monitor the system behavior, united with the technical ability of monitor their values and, moving backward, find out the related mechanistic explanations.

This roadmap outlines a pathway for potentially developing minimal chemical systems with self-regulation, adaptability, and sense-making potential. While distinct from conventional robotics and AI models, such systems expand the material and conceptual space of cognition, and provide a new foundation for future exploration in artificial cognitive architectures. The operational relevance of these principles is further illustrated in the application scenarios of Section 6, where wetware agents are positioned as complementary to embodied and xenobotic AI.

This developmental trajectory for WNAI can be further articulated into a modular program of experimental and conceptual work. Table 3 summarizes five strategic axes that define the core components of this research program. Each axis identifies key directions for possible implementation, spanning from chemical construction to interaction, autonomy, and emergence. Together, they offer a synthetic vision of WNAI as a transdisciplinary framework for exploring the generation of minimal, adaptive, and environmentally coupled cognitive agents.

**TABLE 3:** Strategic axes for the implementation of Wetware Network-Based Artificial Intelligence (WNAI).

Axis	Focus	Description
Bottom-up chemical construction	Protocellular wetware	Compartmentalized, reticular chemical architectures enabling synthetic organization from non-living matter; alternative to xenobot and embodied AI approaches
Reticular cognitive dynamics	Adaptive chemical networks	Self-organizing autocatalytic structures supporting sensing, pattern recognition, and minimal adaptation
Situated interaction	Structural coupling	Design of protocols enabling reciprocal perturbation between chemical agents and their environment
Formalizing autonomy	Organizational closure	Conceptual tools to define closure in non-biological systems; integration of synthetic biology, complex systems theory, and AI
Oriented emergence	Stimulated dynamics	Strategies for guiding the emergence of autonomous reticular behavior; toward situated sense-making

## 6 Contributions for robotics and AI

Building on the theoretical and experimental foundations outlined above, WNAI plays a complementary role alongside embodied and xenobotic AI, expanding operational capabilities and design possibilities without replacing existing paradigms. This complementary role is expressed in three strategic ways:

1. Bridging operational gaps—enabling autonomy and adaptability in domains where electronics or living matter are unsuitable;
2. Enabling hybrid integration—embedding chemical modules within existing robotic platforms to augment sensing, regulation, or decision-making;
3. Opening a new neuromorphic frontier—grounding computation in chemical networks, offering a molecular-scale complement to electronic architectures.

The following scenarios—largely speculative at this stage and representing potential outcomes of later research and development—illustrate how these complementarities could be practically implemented, translating WNAI’s principles into tangible technological pathways in robotics and AI.

### 6.1 Microrobotics for precision medicine

*Current challenges*–Clinical microrobots have demonstrated capabilities in navigation and targeted therapeutic delivery in complex biological environments such as biofilms and microtumors (Zhang et al., 2021; Akolpoglu et al., 2022). However, they still face limitations in autonomy, chemical sensing/processing integration, and independence from external control, which restrict operational effectiveness *in vivo* (Dillinger et al., 2021; Yang et al., 2022; Li et al., 2023).

*Potential WNAI solution*–WNAI could enable embedded chemical-based control architectures capable of interpreting and responding to local biochemical cues without continuous external intervention, allowing real-time adaptation to heterogeneous microenvironments.

*Competitive advantage*–WNAI could support greater autonomy, improved responsiveness to chemical microenvironments, and enhanced operational safety, addressing key barriers to clinical translation.

Supplementary Appendix A presents further reflections on synthetic cells framed within this medical scenario, which currently inspires several authors.

### 6.2 Soft robotics in extreme environments

*Current challenges*–Soft robots can navigate complex, dynamic environments, but extreme conditions such as high salinity, electromagnetic interference, and rapid temperature shifts can degrade performance. These stresses corrode materials, disrupt electronics, and shorten operational lifespans (Hawkes et al., 2017; Lu et al., 2018; Zuniga and Fink, 2023). Even with new materials and hybrid designs (Xu and Gracias, 2019; Yirmibeşoğlu et al., 2019), maintaining full functionality without heavy protective measures remains difficult.

*Potential WNAI solution*–WNAI’s substrate-sensitive chemical controllers could replace or supplement vulnerable electronics, using reaction–diffusion and self-regulating chemical networks that tolerate salinity, temperature variations, and electromagnetic noise.

*Competitive advantage*–WNAI could extend soft robotics into domains such as deep-sea exploration, high-EM industrial zones, and extraterrestrial missions, enabling sustained autonomy without complex shielding.

### 6.3 Ecological monitoring and remediation

*Current challenges*–Remote and sensitive ecosystems require long-term monitoring and remediation with minimal disturbance. Conventional sensor networks need frequent servicing, which is logistically difficult and ecologically disruptive (Matson et al., 2021; Oppong et al., 2023; Laneve et al., 2024). Environmental extremes like biofouling, temperature swings, and limited energy supply further limit their reliability.

*Potential WNAI solution*–WNAI-based chemical sensor–actuator nodes could operate without continuous electronic power, detecting local chemical/biological changes and triggering remediation directly *in situ*. Integrated sensing and actuation in the same substrate would minimize maintenance.

*Competitive advantage*–WNAI could enable self-sustaining, minimally invasive monitoring systems with extended operational lifetimes, preserving ecosystem integrity while providing actionable data.

## 7 Outlooks

*Wetware Network-Based Artificial Intelligence* (WNAI) builds on the legacy of network cybernetics and bottom-up SB to advance a conceptual paradigm and a research program for developing minimal autonomous cognitive agents based on synthetic chemical networks. It reframes cognition as a process emerging from reticular self-organization and material embodiment beyond neural or biological substrates, and outlines testable strategies and modelling pathways for their realization within wetware platforms.

Positioned alongside hardware-based embodied AI and biologically derived xenobotics, WNAI expands the scope of embodiment beyond morphology and evolutionary substrates, grounding it in the self-organizing dynamics of chemical networks. Our perspective, that we have described as based on SB and systems chemistry methodologies, differs epistemologically (but it might coincide operationally) with others chemical computing approach. Examples of the latter are quantum computing, DNA computing, and molecular circuits.

Quantum computing uses qubits that exploit superposition and entanglement to perform computations far beyond classical capabilities. It promises breakthroughs in cryptography, optimization, and materials science. It has been proposed that quantum computing will allow solving problems intractable for classical systems, such problems with exponential complexity and probabilistic outcomes (Sharma and Saxena, 2025). DNA computing, such as strand displacement and DNA origami, harnesses biochemical reactions and the structure of DNA molecules to perform computations in massive parallelism. DNA computing encodes information in nucleotide sequences and performs logic operations via hybridization, ligation, and enzymatic reactions, expanding its computational versatility in molecular programming (Xu et al., 2025). Molecular circuits use individual molecules or nanoscale assemblies, representing a frontier in miniaturization, enabling ultra-small, energy-efficient devices. These circuits can mimic traditional components like transistors, switches, diodes, and logic gates, potentially revolutionizing electronics beyond the limits of silicon. They can be suitable for ultra-low-power embedded computation and integration into molecular-scale devices (Sun et al., 2014; Huang and Li, 2020).

What makes WNAI innovative, and in a certain sense encompassing several chemical approaches, is the explicit reference to define a conceptual space where artificial chemical networks of different types (thus, including those listed above) are designed and conceived to be epistemologically convergent realizations of autonomous cognition. In other words, the emphasis is shifted from a substrate neuromimicry aimed at building forms of computations (more powerful or more versatile than conventional computational architectures) to network-based autonomy, and from substrate imitation—i.e., neuron imitation—to a broader set of synthetic implementations (constructions). While the latter aspect, the synthetic one, is also shared by the above-mentioned chemical computing approaches, our proposal avoids the conventional interpretation of computation based on representations and their manipulations, preferring instead to focus on the autonomous regulation of systemic processes, subordinated (constrained) to the maintenance of the whole. If the term computation is to be employed within an autopoietic or autonomous framework, it should be reconceptualized as auto-computation—computation enacted by, upon, and in service of, the system’s functioning itself.

Looking ahead, the research agenda will focus on identifying the conditions under which chemical systems exhibit reticular closure, sustained dynamical regulation, and environmentally responsive behaviour. The goal is not to compete with general-purpose AI architectures, but to develop novel forms of artificial cognition adapted to domains where wetware agents offer distinctive modes of adaptability and regulation—such as microrobotics for medicine, soft robotics in extreme environments, and ecological monitoring. In these contexts, chemical embodiment provides operational strategies inaccessible to silicon-based systems while opening new opportunities for minimal, adaptive, and socially sustainable intelligence. As a *Hypothesis and Theory* contribution, this article provides a coherent framework and testable research agenda intended to stimulate and orient future work in this important direction for the communities engaged in robotics and AI, particularly in the development of robotic systems and artificial intelligence architectures.

Ultimately, WNAI suggests expanding the scope of cognitive science and AI by acknowledging the constitutive role of material organization. Such an expansion opens experimental domains—from prebiotic chemistries to morphogenetic dynamics and ecologically embedded environments—where cognition is enacted rather than programmed, and where chemical embodiment enables operational strategies inaccessible to conventional systems. These systems can be considered as forms of artificial “diverse intelligence” (Levin, 2025), and possess the ability to generate meaning, solve problems, and make decisions beyond the conventional realm of Boolean logic of computationalist models. Rather than simply mimicking traditional computational paradigms, they operate across multiple domains, including metabolic, morphological, and physiological spaces. In essence, they function within a network of reticular processes acting creatively and adaptively, yet constrained by physico-chemical determinants and by their inherent organizational structures.

## Data Availability

The original contributions presented in the study are included in the article/Supplementary Material, further inquiries can be directed to the corresponding author.
