# Supplementary material for: Wetware network-based AI: a chemical approach to embodied cognition for robotics and artificial intelligence
Source: Front Robot AI. 2026 Jan 5;12:1694338. doi: 10.3389/frobt.2025.1694338 (PMC12812610; doi:10.3389/frobt.2025.1694338)
Supplement: Supplementary file 1 [file Supplementaryfile1.docx]

Supplementary Material

# Supplementary Appendix A

To complement the contents of this article, this appendix expands on the biomedical microrobotics scenario, a domain where WNAI principles may find early application. In this context, the field of micro- and nanorobots represents a particularly relevant area of medical robotics, focused on the design, programming, production, and management of robots at the micro- and nanoscale.

The rapidly progressing technology of synthetic cells is attracting the attention from specialists in that area, because synthetic cells might offer selective advantages, in specific applications, when compared to other micro- and nanoparticles-based systems. The potential applications are multifold: targeted or controlled drug delivery, tissue engineering, biosensing/imaging, red blood cell biomimicry, anaemia treatment, biofilm digestion, viral removal, biological sensing, detoxification, cardiovascular diseases, etc. (Ren et al., 2024; Zarepour et al., 2024; Kumar et al., 2025). Nanomedicine, that born with liposomal drug delivery (Lasic and Papahadjopoulos, 1998), can further evolve thanks to this new biotechnology, and become “smart”, cognitive, and adaptive. Scaling-up challenges (Sharf-Pauker et al., 2025), public acceptance (Rook et al., 2025) and regulation needs (Sampson et al., 2024) have been discussed too.

Detailed discussions, primarily in the form of perspectives and hypotheses, though with some notable exceptions (Krinksy et al., 2018; Toparlak et al., 2020; Chen et al., 2022), can be found in dedicated publications (Sato et al., 2022; Sümbelli et al., 2023; Waeterschoot et al., 2024; Kim et al., 2025; Maia et al., 2025; Yandrapalli, 2025).

It is worth recalling that the construction of synthetic cells for nanomedical applications can, within a computationalist paradigm, be interpreted as the creation of chemical robots equipped with a form of chemical AI. This perspective—common in bioengineering and SB—also intersects with research in molecular communication (Hiyama et al., 2005; Nakano et al., 2013) and the emerging field of the Internet of Bio-Nano Things (IoBNTs) (Akyildiz et al., 2015; see commentary in Stano et al., 2023).

Biomedical micro-robotics provides one illustrative domain where WNAI principles may be explored, alongside other possible applications. In continuity with the main text, here we have highlighted how wetware approaches can inspire concrete scenarios within robotics and AI.

# Supplementary References

Akyildiz, I. F., Pierobon, M., Balasubramaniam, S., and Koucheryavy, Y. (2015). The internet of Bio-Nano things. IEEE Communications Magazine 53, 32–40. doi: 10.1109/MCOM.2015.7060516

Chen, G., Levin, R., Landau, S., Kaduri, M., Adir, O., Ianovici, I., et al. (2022). Implanted synthetic cells trigger tissue angiogenesis through de novo production of recombinant growth factors. Proc Natl Acad Sci USA 119, e2207525119. doi: 10.1073/pnas.2207525119

Hiyama, S., Moritani, Y., Suda, T., Egashira, R., Enamoto, A., Moore, M., et al. (2005). “Molecular communications,” in Technical Proceedings of the 2005 NSTI Nanotechnology Conference and Trade Show (Anaheim, 8-12 May 2005), TechConnect Briefs, 391–394.

Kim, W., Han, J., Chauhan, S., and Lee, J. W. (2025). Cell-free protein synthesis and vesicle systems for programmable therapeutic manufacturing and delivery. J Biol Eng 19, 55. doi: 10.1186/s13036-025-00523-x

Krinsky, N., Kaduri, M., Zinger, A., Shainsky-Roitman, J., Goldfeder, M., Benhar, I., et al. (2018). Synthetic Cells Synthesize Therapeutic Proteins inside Tumors. Adv Healthc Mater 7, e1701163. doi: 10.1002/adhm.201701163

Kumar, P. V., Tian, F. K., Le Yi, C., Natarajan, E., Bin, L. K., and Karahan, M. (2025). Micro- and nanorobots for pioneering precision medicine and medical processing at the cellular level. Int J Intell Robot Appl. doi: 10.1007/s41315-025-00471-1

Lasic, D. D., and Papahadjopoulos, D. (Editors) (1998). Medical Applications of Liposomes. Amsterdam: Elsevier.

Maia, R. F., Vaziri, A. S., Shahbazi, M.-A., and Santos, H. A. (2025). Artificial cells and biomimicry cells: A rising star in the fight against cancer. Mater Today Bio 32, 101723. doi: 10.1016/j.mtbio.2025.101723

Nakano, T., Eckford, A. W., and Haraguchi, T. (2013). Molecular Communication. Cambridge UK: Cambridge University Press.

Ren, Z., Wan, Q., Zhu, Y., Li, L., Wang, K., Zhao, F., et al. (2024). Atypical artificial cells: Novel biomimetic materials for combating cancer. Interdisciplinary Materials 3, 658–714. doi: 10.1002/idm2.12199

Rook, O., Zwart, H., and Dogterom, M. (2025). Public attitudes to potential synthetic cells applications: Pragmatic support and ethical acceptance. PLoS One 20, e0319337. doi: 10.1371/journal.pone.0319337

Sampson, K., Sorenson, C., and Adamala, K. P. (2024). Preparing for the future of precision medicine: synthetic cell drug regulation. Synth Biol (Oxf) 9, ysae004. doi: 10.1093/synbio/ysae004

Sato, W., Zajkowski, T., Moser, F., and Adamala, K. P. (2022). Synthetic cells in biomedical applications. Wiley Interdiscip Rev Nanomed Nanobiotechnol 14, e1761. doi: 10.1002/wnan.1761

Sharf-Pauker, N., Galil, I., Kfir, O., Chen, G., Menachem, R., Shklover, J., et al. (2025). Scaling Up Synthetic Cell Production Using Robotics and Machine Learning Toward Therapeutic Applications. Adv Biol (Weinh) 9, e2400671. doi: 10.1002/adbi.202400671

Stano, P., Gentili, P. L., Damiano, L., and Magarini, M. (2023). A Role for Bottom-Up Synthetic Cells in the Internet of Bio-Nano Things? Molecules 28, 5564. doi: 10.3390/molecules28145564

Sümbelli, Y., Mason, A. F., and van Hest, J. C. M. (2023). Toward Artificial Cell-Mediated Tissue Engineering: A New Perspective. Adv Biol (Weinh) 7, e2300149. doi: 10.1002/adbi.202300149

Toparlak, Ö. D., Zasso, J., Bridi, S., Serra, M. D., Macchi, P., Conti, L., et al. (2020). Artificial cells drive neural differentiation. Sci Adv 6, eabb4920. doi: 10.1126/sciadv.abb4920

Waeterschoot, J., Gosselé, W., Lemež, Š., and Casadevall I Solvas, X. (2024). Artificial cells for in vivo biomedical applications through red blood cell biomimicry. Nat Commun 15, 2504. doi: 10.1038/s41467-024-46732-8

Yandrapalli, N. (2025). Bottom-up development of lipid-based synthetic cells for practical applications. Trends Biotechnol, S0167-7799(25)00094–0. doi: 10.1016/j.tibtech.2025.03.009

Zarepour, A., Khosravi, A., Iravani, S., and Zarrabi, A. (2024). Biohybrid Micro/Nanorobots: Pioneering the Next Generation of Medical Technology. Advanced Healthcare Materials 13, 2402102. doi: 10.1002/adhm.202402102
